# Supplementary material for: Rapid discovery of new-to-nature protein domains by novelty-first forcing of language models
Source: bioRxiv. 2025 Oct 2:2025.10.02.679910. Preprint. [Version 1] doi: 10.1101/2025.10.02.679910 (PMC12622050; doi:10.1101/2025.10.02.679910)
Supplement: 1 [file NIHPP2025.10.02.679910V1-supplement-1.pdf]

bioRxiv preprint doi: https://doi.org/10.1101/2025.10.02.679910; this version posted October 2, 2025. The copyright holder for this preprint (which was not certified by peer review) is the author/funder, who has granted bioRxiv a license to display the preprint in perpetuity. It is made available under aCC-BY 4.0 International license.

4 Supplemental Material

4.1 Methods

4.1.1 Fragment Library Assembly for Genetic Algorithm

The initial fragment library for the structure discovery genetic algorithm was assembled via a replica-exchange Markov Chain Monte Carlo (RE-MCMC) approach for sampling over a sequence landscape. We construct a two-term energy function that combines a preference for accepting mutations that increase sequence-likelihood and a preference for increasing predicted structural contact density, metrics that are inferred from the ESM2-650M model [Lin et al., 2023]. For a *single chain*: if an individual sequence s_i of length N has an associated log-likelihood $L_i = \prod_{k=1}^{N} l_k$ where the l_k represent independent residue-wise likelihoods, we can write ΔE for a proposed move from sequence s_i to s_j as

$$\Delta E_{i \to j} = (\log L_j - \log L_i) + w_c \frac{1}{n^2} (\sum_{kl} C_{j,kl} - \sum_{kl} C_{i,kl}) \tag{1}$$

where n is the fixed sequence length, C_i, C_j are binary contact matrices s.t. $C_{i,kl} = 1$ indicates that residues k and l of sequence i are predicted to be in physical contact within < 8 Åin the corresponding three-dimensional structure, and w_c controls the relative weights of the likelihood and contact terms. Contact matrices are inferred from the contact_prediction head of ESM2-650M, simultaneously with log-likelihood calculation. We restrict allowable moves to single point mutations, though the formulation holds generally for any s_i, s_j of the same length.

The standard Metropolis-Hastings criterion is used, setting the acceptance probability for a proposed single point mutation (sampled uniformly across sequence positions and amino-acid identities) from s_i to s_j for a *single chain* to

$$p_{i \to j} = \min\{1, \exp(\beta \Delta E_{i \to j})\} \tag{2}$$

For RE-MCMC, several chains are monitored simultaneously following the above, sampling the same landscape at different temperatures, thereby balancing riskier less-local moves by "hot" chains with more conservative local moves by "cold" chains. Adjacent chains in the temperature array attempt to swap positions on the landscape (and their respective sequences) periodically at a stochastic frequency λ; the proposed swap move between chains i, j is accepted with probability

$$p_{i \leftrightarrow j} = \min\{1, \exp[(E_i - E_j)(\beta_i - \beta_j)]\} \tag{3}$$

where $\{\beta_i\}$ refers to thermodynamic β, the inverse of the sampling temperature T.

A total of 800 fragments were generated, running for $n = 5000$ steps, stochastically attempting to swap a uniformly randomly selected pair of adjacent random chains at a rate of $\lambda = 0.01$ swp/step, 5 chains with inverse temperatures $\beta = \{20, 13.\bar{3}, 10, 8, 6.\bar{6}\}$ from "cold" to "hot," and $w_c = 1$. Initial sequences for all chains $\{s_0\}$ were random amino-acid strings (sampled uniformly with respect fo amino-acid identity) of length 40; the coldest chain ($\beta = 20$) sequence at step 5000 was added to the library.

4.1.2 Structure Discovery Genetic Algorithm

The structure discovery genetic algorithm begins by sampling an initial population P_0 of 100 fragments from a fragment library assembled as described. For a fixed number of rounds, the k-th round proceeds by:

1. Generating 20 new variants from P_{k-1}. A pair of variants is generated by drawing two sequences uniformly at random from P_{k-1}, performing a crossover operation with the number of crossover points $n_{cross} \sim$ Poisson($\lambda = 1.535$) and the locations of the crossover points uniformly distributed over the sequence length(s), and performing a mutation operation with the number of mutations $n_{mut} \sim$ Binom($n_i, \lambda = 0.05$) and mutation locations and identities uniformly distributed over sequence lengths.

bioRxiv preprint doi: https://doi.org/10.1101/2025.10.02.679910; this version posted October 2, 2025. The copyright holder for this preprint (which was not certified by peer review) is the author/funder, who has granted bioRxiv a license to display the preprint in perpetuity. It is made available under aCC-BY 4.0 International license.

2. Evolving the new (k)-th round variants through eeMHMC with the energy function found in Eq. 1 for $n = 5000$ steps, with $w_c = 1$, $\beta = 10$, and adaptive temperature adjustment at 100-step intervals.
3. Adding the evolved variants to P_{k-1} to form P_k.
4. Predicting structures and computing the amino-acid surface-area burial fraction for *all* sequences in P_k.
5. Stochastic selection for burial fraction, maintaining a target constant population size of 100.

The above procedure repeats up to a desired number of generations (200 in this study). To enforce constant population size while stochastically eliminating sequences from the population, we note that:

If the number of surviving sequences after round k is to be $|P_k| = N_{sel}$, then the expectation of N_{sel} must be

$$E[N_{sel}] = Nf_{sel} \tag{4}$$

where N is the temporary population size after new variants have been added but before any have been removed, and f_{sel} is the fraction of sequences that are to survive. We can additionally write that

$$E[N_{sel}] = \sum_i E[n_i] = \sum_i \Pr(\Theta_i = 1) \tag{5}$$

where $\Theta_i \sim \text{Bernoulli}(p_i)$ for some mathematically appropriate p_i, as the survival of a given sequence is independent of the survival probability of all others. We have the choice of the form of p_i and so take $p_i = \exp[-\beta_k(0.8 - \gamma_i)]$, where β_k is a sampling hyperparameter to be determined and γ_i is the burial fraction of sequence s_i

$$Nf_{sel} = E[N_{sel}] = \sum_i E[n_i] = \sum_i \exp[-\beta_k(0.8 - \gamma_i)] \tag{6}$$

Note that if $\gamma_i > 0.8$, then this would imply $p_i > 1$; formally, we ought to say $p_i = \max\{1, \exp[-\beta_k(0.8 - \gamma_i)]\}$; empirically, however, the burial fraction for even exceptionally well-packed and folded protein domains is bounded above by $\gamma_i = 0.8$.

Making the simplifying assumption that the $\{\gamma_i\}$'s are roughly normally distributed – empirically justified for ESM2-generated sequence and reasonably extrapolated to sequences being evolved subject to an ESM2-based energy function – we can say

$$Nf_{sel} = E[N_{sel}] = \sum_i E[n_i] \approx N \exp[-\beta_k(0.8 - \bar{\gamma}_i)] \tag{7}$$

where $\bar{\gamma}_i$ is the mean of all calculated burial fractions in the temporarily augmented population P_k leaving only algebraic rearrangement to solve for our lone sampling hyperparameter β_k, effectively a selection inverse temperature, as follows

$$\beta_k = \frac{-\log f_{sel}}{0.8 - \bar{\gamma}_i} \tag{8}$$

This completes the material necessary to specify and implement the structure discovery genetic algorithm.

4.1.3 Structure-First Foldtuning

Foldtuning was performed and implemented essentially as described in Subramanian et al. [2025] with the following modifications: (1) generation of 10,000 sequences per round in batches of 250; (2) selection of sequences satisfying structural compactness (amino-acid surface burial fraction > 0.5) and novelty (no CATHDB50 hit with TMscore > 0.5) criteria; (3) ranking of filtered, validated round n sequences for round $n + 1$ finetuning in descending order of amino-acid surface burial fraction; (4) omission of the initial "evotuning" round due to absence of a specific target fold.

bioRxiv preprint doi: https://doi.org/10.1101/2025.10.02.679910; this version posted October 2, 2025. The copyright holder for this preprint (which was not certified by peer review) is the author/funder, who has granted bioRxiv a license to display the preprint in perpetuity. It is made available under aCC-BY 4.0 International license.

4.1.4 Selection of Novel Folds for Computational Characterization

For the genetic algorithm experiment, all fifteen putative novel folds were advanced to the computational validation and characterization. For the foldtuning-based experiment, 1018 putative novel folds were initially cumulatively identified over five rounds of structure-first foldtuning. To remove redundancy, predicted structures of the 1018 were clustered with FOLDSEEK at a similarity threshold of TMscore $= 0.5$, decreasing the number of templates to 916. Given that the structural diversity of the whole AlphaFoldDB50 runs deeper than that of the CATHDB50 subset, the 916 remaining putative novel folds were searched, again using FOLDSEEK, against the entire AlphaFoldDB50, dropping structures with any single hit with alignment region TMscore > 0.5. This reduced the number of templates to 762. These 762 templates were ranked in order of decreasing surface-area burial fraction and the top 100 carried through for inverse-folding and energy scoring validation. For Fig. 4 and Fig. 5, only the further top 10 of these top 100, as ranked by lowest (most-stable) mean Rosetta-scored energy over all inverse-folded sequences are depicted.

4.1.5 Structure Prediction and Assignment

All structures were predicted with default ESMFold inference parameters as in Lin et al. [2023]. Predicted structures were annotated to CATH domain labels via FOLDSEEK structure-based search against the prebuilt CATHDB50 database of Lau et al. [2024], running in accelerated TMalign mode. The consensus CATH domain was defined as the fold accounting for the most hits with TMscore > 0.5 and max(query_coverage, target_coverage) > 0.8. In the absence of at least one hit satisfying these criteria, a structure was considered to be un-assignable.

4.1.6 Basic Chemical Property Calculations

Amino-acid surface area burial fraction was calculated using custom code and reference individual amino-acid surface areas (HMS Bionumbers: 103239).

4.1.7 Energy Scoring Calculations

Biomolecule energy scores were obtained using the default 'ref2015' energy function and standard relaxation and scoring workflow in ROSETTA v3.11, as described in Alford et al. [2017]. Energy scores are reported in **R**osetta **E**nergy **U**nits (R.E.U.), normalized to sequence length.

4.1.8 Validation of Inverse-Folding Sequences and Structures

For both the genetic algorithm and foldtuning-based experiments, 200 sequences were generated per structural template with ProteinMPNN, using the vanilla—v_48_020 model, sampling temperature 0.2, Gaussian backbone noise with per-coordinate $\sigma = 0.1$ Å, and forced omission of the rare/ambiguous amino acids B, J, O, U, X, and Z [Dauparas et al., 2022]. Within each batch of 200, sequences were downclustered at 60% sequence identity with MMSEQS2, structures predicted with ESMFold, and queried against the template structure with FOLDSEEK in TMalign mode using the standard TMscore > 0.5 threshold as confirmation of a global match.

4.2 Supplemental Figures

bioRxiv preprint doi: https://doi.org/10.1101/2025.10.02.679910; this version posted October 2, 2025. The copyright holder for this preprint (which was not certified by peer review) is the author/funder, who has granted bioRxiv a license to display the preprint in perpetuity. It is made available under aCC-BY 4.0 International license.

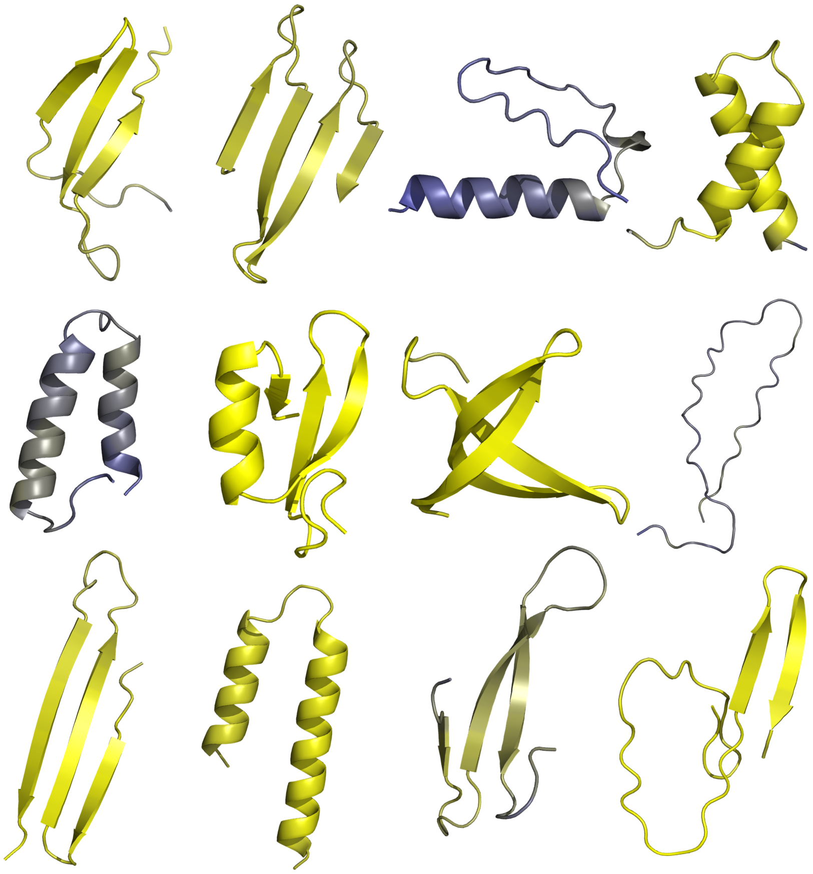

Figure S.1: **Example structure fragments generated by RE-eeMHMC.** 10 of 800 structure fragments predicted from sequences designed by replica-exchange explore-exploit Metropolis-Hastings Monte Carlo sampling (RE-eeMHMC). Individual structures are colored by ESMFold pLDDT; yellow=high, blue=low.

bioRxiv preprint doi: https://doi.org/10.1101/2025.10.02.679910; this version posted October 2, 2025. The copyright holder for this preprint (which was not certified by peer review) is the author/funder, who has granted bioRxiv a license to display the preprint in perpetuity. It is made available under aCC-BY 4.0 International license.

4.3 Supplemental Tables

Table S.1: **Metadata and structural alignment metrics for closest CATH domain Foldsheek hits to 15 novel folds proposed by the genetic algorithm approach.**

	Closest CATH Hit					
Novel Fold	ID	Name	PDB/AFDB	Pos.	TM	RMSD (Å)
0CF85E_R97	2.60.40.60	Cadherins	0Q7TSF1	383-484	0.428	7.7
120FD5_R140	1.20.5.4130	n/a	A0A0P0YA47	9-126	0.351	5.8
244D7D_R143	1.10.533.10	Death Domain, Fas	Q4QQS0	2-94	0.491	4.1
26D32B_R192	2.40.160.200	LURP1-related	A0A0K3ARQ4	139-303	0.500	6.3
3733B8_R10	2.30.30.170	n/a	Q2FZK7	945-1013	0.430	6.5
794026_R125	1.10.8.430	Helical domain of apop...	Q6Z392	364-452	0.319	7.8
9D1265_R55	1.25.40.10	Tetratricopeptide repeat...	Q9LEX5	342-409	0.540	3.0
A0A7B8_R123	1.10.472.10	Cyclin-like	F4IWI9	175-2664	0.368	7.7
A49A4F_R116	1.10.260.40	λ repressor-like DNA-bind...	1ic8A	87-180	0.319	5.4
A78532_R160	1.20.140.150	n/a	Q7YTM8	1-160	0.396	5.1
B4RC4F_R164	3.90.1150.210	F-actin capping protein...	3aa7B	90-244	0.422	6.9
BC29B7_R55	1.10.357.10	Tet repressor, domain 2	1Z77A	47-200	0.430	6.7
C86FA9_R143	3.30.1520.10	Phox-like domain	Q54S15	808-935	0.477	6.0
DB6817_R173	1.10.520.10	n/a	K7VNV5	33-159	0.393	5.6
F99539_R114	1.10.10.60	Homeodomain-like	1ic8B	203-276	0.473	4.1

Table S.2: **Metadata and structural alignment metrics for closest CATH domain Foldsheek hits to 10 novel folds proposed by structure-first foldtuning.**

	Closest CATH Hit					
Novel Fold	ID	Name	PDB/AFDB	Pos.	TM	RMSD (Å)
5_111	1.10.472.10	Cyclin-like	I1M2D8	39-142	0.514	4.6
5_4773	1.10.10.10	Winged helix DNA-bind...	Q2FWL6	1-80	0.392	11.8
5_4799	1.10.472.10	Cyclin-like	Q10QA2	94-195	0.422	9.1
4_2316	1.10.533.10	Death Domain, Fas	F8VQ39	371-466	0.479	7.5
2_385	1.10.150.130	Tyr recombinase, N-term...	2keyA	1-112	0.526	4.4
3_8774	1.10.472.10	Cyclin-like	P51946	41-159	0.459	5.2
4_6556	1.20.920.10	Bromodomain-like	A0A1I9LTJ3	289-399	0.402	5.4
4_6411	1.20.960.30	Mitochondrial import rec...	1uujA	2-77	0.419	3.4
3_5721	3.30.980.10	Threonyl-trna synth...	Q9VUJ0	131-293	0.309	5.0
2_3053	1.10.10.1440	PHAX RNA-bind...	2xc7A	1-104	0.398	5.6
